# The Bacillus of Abortion

**Published:** 1884-10

**Authors:** 


The Bacillus of Abortion.—Among all the theories which have been advanced to explain the cause of abortion in cows, that which supposes the existence of a micro-organism deserves now the most attention. We refer of course to that form of abortion which occurs as an enzootic or epidemic. The natural history of the trouble, when it attacks herds in this way, points to the existence of some living organism as the etiological factor. The disease attacks a herd, and spreads gradually through it, lasting for two or three years. There seems to be a kind of incubation period after a cow has been exposed. Careful isolation and disinfection lessen the spread of the disease. The abortions are undoubtedly caused by some general endemic influence. Pathology teaches us that in these cases there is pretty surely a bacillus present. The theory that abortion is a purely nervous trouble, and that cows abort through sympathy, is strained, and not in harmony with the known stolidity of the bovine nervous system.

Too much has been written and too much imagination has been expended already upon this subject; therefore we shall go no further into detailed argumentation.

We are seriously impressed, however, with the need of careful investigation of this subject with the modern appliances of mycological research. A little skilled effort in this direction could answer the question as to a bacillary origin of abortion, with absolute certainty. Let our Government or our colleges institute a search for the bacillus of abortion. We need hardly add that this latter parasite, if found, would become the darling of a large class of American women! C.

